# Power Spectrum Features of Acupoint Bioelectricity Signal

**DOI:** 10.1155/2021/6638807

**Published:** 2021-04-12

**Authors:** Jingjing Zhang, Renhuan Yu, Enlu Zhao, Quan Zhou, Shuping Gai

**Affiliations:** ^1^Department of Nephrology, Xiyuan Hospital of the China Academy of Chinese Medical Sciences, 1 Xiyuan Caochang, Haidian District, Beijing 100091, China; ^2^State Key Laboratory of Transducer Technology, Institute of Electronics, Chinese Academy of Sciences, 19 Fourth Ring Road North, Haidian District, Beijing 100090, China

## Abstract

**Background:**

Since the 1950s, many studies have been conducted on the electrical properties of acupuncture points (acupoints), especially their bio-resistance characteristics. Results of such studies have been inconclusive due to factors such as sweat gland density and compounding factors of applying electrical stimulation. In this study, a power spectrum instrument was used to assess the power spectrum and power of acupoints and nonacupoints without electrical stimulation. Using such instrumentation, specificity of electrical signals of acupoints was also explored.

**Methods:**

Thirty-six subjects (29 females, 7 males) participated in the study. Stainless steel acupuncture needles (diameter 0.35 mm; length 50 mm) were used. Five acupoints were tested: ST 36, SP 6, GB 39, GB 37, and K I9. Four control sites 0.5–1.0 cm adjacent to each acupoint were chosen. After needle insertion into the acupoint and control sites, the needles were attached to the power spectrum instrument to acquire any electrical signals. Acquire signals were analyzed using self-written software.

**Results:**

Power spectrum difference between acupoint and nonacupoint signals was 0–2 Hz. Results of *t*-test or signed rank sum test (*α* = 0.05) found that electrical signals between acupoints and nonacupoints were markedly different (*P* < 0.05).

**Conclusion:**

Acupoint bioelectricity signals are higher than adjacent nonacupoints. The most significant difference is distributed between 0 Hz and 2 Hz.

## 1. Background

A large volume of research has been conducted on acupuncture points (acupoints) and meridians (channels), focusing on the physiologic effects of acupoints and their electrophysiologic characteristics. The therapeutic effect of acupuncture has been confirmed by numerous clinical trials, showing that specificity of a given acupoint is associated with its efficacy. For example, acupuncture applied at ST 2, ST 25, and ST 36 increases gastric antral volume [1]. Copious randomized controlled trials have found treating acupoints is efficacious over treating nonacupoints (sham) [2]. However, studies have also shown no therapeutic differences between acupoints and nonacupoints [3, 4].

Since the 1950s, many studies have been conducted on the electrical properties of acupoints, especially their bio-resistance characteristics [5–7]. Such studies have suggested that acupoints maybe the particular channel transmitting electrical signals, compared with nonacupoints; acupoints can enhance conductivity, reduce electrical resistance or impedance, and increase electrical potential [8–10].

Many factors affect acupoint resistance properties, such as sweat gland density, acupoint location, electrode polarization, and frequency, among others [11]. To overcome these factors, Gow et al., for example, used a Kelvin probe that circumvented skin contact to detect acupoint resistance. However, applicability of this method may be limited as the probe is sensitive to ambient field and movement artifacts [12].

Instead, we propose that applying technology similar to electrocardiography or electroencephalography may detect the true nature of the electrical signal of acupoints. In this study, power spectrum instrumentation was used to collect electric signals at acupoints and nonacupoint control locations. External electrical stimulation was not applied, allowing better understanding of the difference, if any, between signals found at acupoints and nonacupoints, and specificity of the electrical signals.

## 2. Materials and Methods

### 2.1. Subjects

Thirty-six subjects (29 female, 7 male) between 23 and 30 years old were recruited to participate in the study during February–July 2018. Participants were recruited in Xiyuan Hospital of China Academy of Chinese Medical Sciences.

Subjects were excluded if they were under 18 years old and pregnant, had clear signs and symptoms of disease, and were suspected of having significant organic disease, lactating, or menstruating. On the day of the study, volunteers were queried about their previous day's emotional and dietary circumstances and those who experienced excess emotions (such as a bout of anger), ate cold or spicy food, or over-ate were excluded. Testing took place in the Nephrology Department of Xiyuan Hospital.

### 2.2. Acupoints Tested

**Figure 1:**
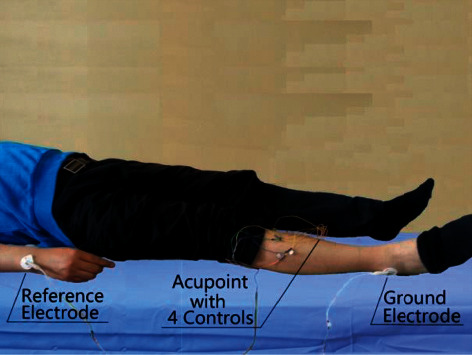
Reference and ground electrode sites.

Acupoints tested were located on the right side: ST 36, SP 6, GB 37, GB 39, and KI 9. Nonacupoint control points were situated 0.5–1.0 cm around the tested acupoints. The signal acquisition instrument required a reference electrode. A nonacupoint site on the right ulnar styloid process was chosen because of its limited number of sweat glands, allowing for voltage stability. A grounding electrode was also required, and the apex of the right lateral malleolus was selected for attachment (Figure 1).

### 2.3. Experimental Device

**Figure 2:**
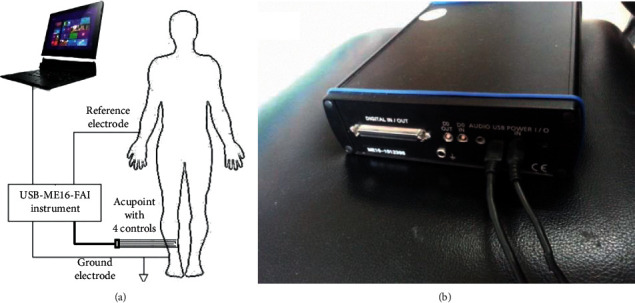
(a) Connections between computer and signal acquisition instrument and the subject. (b). USB-ME16-FAI signal acquisition instrument.

The study was conducted with a USB-ME16-FAI System (Multichannel Systems, Reutlingen, Germany), a highly sensitive electrical signal acquisition instrument (Figure 2). We wrote a MATLAB (MathWorks, Natick, MA) program to analyze the power spectrum of acquired signals.

### 2.4. Experimental Procedure

The experiment was performed in a quiet environment with minimal air flow and an ambient temperature of 18°C–24°C. Participants were shielded from any electromagnetic radiation.

Participants were asked to arrive 10 minutes before the start of the experiment so that they could sit quietly and relax. They were then asked to lie supine on a treatment table with the right upper arm and right lower leg exposed. Participants were also asked to stay awake during the experiment. In order to ensure the accuracy of the test, a single practitioner was responsible for a single acupoint.

To ensure good skin contact, the reference and ground electrodes were cleaned with 75% isopropyl alcohol before attachment to the skin. Disposable electrode pads were supplied by Beijing Tianhe Weiye Medical Equipment Factory (Beijing, China). The electrodes were then connected to the signal acquisition instrument with lead wires. The five acupoints were tested individually: ST 36, SP 6, GB 39, GB 37, and K I9. Four nonacupoint sites were selected to serve as controls. They were located 0.5–1.0 cm lateral, medial, superior, and inferior to each acupoint. These areas were cleaned with 75% isopropyl alcohol.

**Figure 3:**
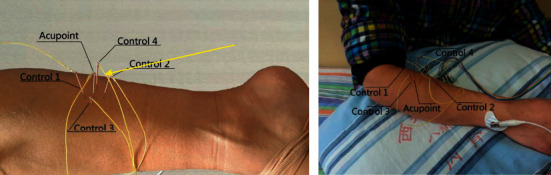
Placement of needles at acupoint and 4 surrounding control points.

Off-the-shelf disposable steel acupuncture needles (Suzhou Huanqiu Acupuncture Medical Appliance Co., Ltd., Suzhou, China) were used (diameter 0.35 mm; length 50 mm). The needle at the acupoint was inserted to a depth of 15 mm and *deqi* was obtained. To ensure reliability of the results, insertion depths of acupoint and control point needles were the same, though *deqi* was not apparent at the control points (Figure 3). One end of a lead wire was attached to the needle handle, with the other end attached to the signal acquisition instrument. Bioelectric signals of acupoints and control points were recorded synchronously by the USB-ME16-FAI instrument. Duration of each test was approximately 1 min. After all needles were removed, subjects were advised to rest quietly for 5 min.

We denoised the signals with db5 wavelet. Using Welch's method, power spectral density (PSD) was evaluated. Analyzing the power spectrum of the signal after filtering, the sampling frequency of instrument was 1000 Hz, thus the power spectrum analysis range was to maximum of 500 Hz. To easily observe the specificity, 0–2 Hz, 0–10 Hz, 0–50 Hz, and 0–500 Hz were analyzed.

### 2.5. Statistics

The Shapiro–Wilk normality test was applied if the data were consistent with a normal distribution based on the *t*-test. If the data did not meet normal distribution, signed rank sum test was used. Differences between acupoints and nonacupoints were considered significant if *P* < 0.05 or less.

## 3. Results

**Figure 4:**
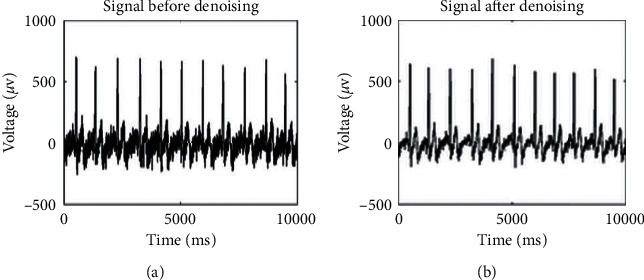
Typical signals from right leg. (a). Before denoising. (b). After denoising.

Five acupoints and their associated nonacupoint control points were scanned on 36 participants. Typical signals recorded by the instrument on the right leg are shown in Figure 4.

Analyzing the power spectrum of the signal after denoising, results showed that the difference in power spectrum between acupoint and nonacupoint bioelectrical signals, most of the high power, is distributed in the 0–10 Hz, especially in the 0–2 Hz.

The first statistical analysis showed that the difference between bioelectrical output of acupoints and nonacupoints was significant (*P* < 0.05). ST 36, SP 6, GB 37, and GB 39 were considered different from their control points. With K I9, the data did not meet normal distribution, thus using signed rank sum test, the difference between this acupoint and its control points was significant (*P* < 0.05) (Table 1).

A second statistical analysis assessed all 48 pieces of data using Shapiro–Wilk method, finding that the results did not meet normal distribution. Next, using the *t*-test (alpha 0.05), it was revealed that the difference between acupoint and nonacupoint bioelectrical output was significant (*P* < 0.05) (Table 2).

## 4. Discussion

Numerous studies on the characteristics of acupoints have shown that they do in fact possess bioelectric properties, including increased conductance, reduced impedance and resistance, and increased capacitance. Recent studies have focused on the volt-ampere characteristics of acupoints [13], using electrical impedance methodology [14, 15]. Yet the majority of instruments employed measure electrical skin resistance, which differs from detecting and monitoring acupoints. Controversial findings may be due to different measuring methods. Most studies have focused primarily on the efficacy and electrical characteristics of acupoints and their active response to external electric stimulation. This cannot be equated with acupoints' own bioelectrical properties. We believe that applying signal acquisition methods similar to ECG and EEG testing to detect bioelectrical signals at acupoints may lead to understanding the true nature of the bioelectrical characteristics of acupoints.

So, we designed this experiment to test this idea. From the test results, we can basically affirm that acupoints have certain electrical properties, which are an aspect of the specificity of acupoints. The study of the electrical properties inside acupuncture points should ensure their objectivity and reproducibility, which is of great importance to fully reveal the electrical properties of meridian points.

The instrument used in this experiment, the first of its kind in China, was used to directly collect acupoint bioelectricity signal. The specific details will be explained one by one as follows:

1. Why the acupoints on the right side of the limb were chosen: firstly, to avoid the influence of cardiac electricity, we chose the reference electrode, the grounding electrode, and the test acupoints on the same side of the limb; secondly, we chose all the acupoints on the right side of the limb because of the lying position of the volunteers and the convenience of the acupuncturist. Of course, this is the limitation of this test, and we can select the left limb acupoints for comparison in the future.
2. It was observed that the conductivity of electrical conduction between LI 4 and LI 11 was significantly higher than that between nonacupuncture points 1 cm outside of LI 4 and LI 11. Because of the differences in body size of the volunteers, we chose 0.5–1.0 cm of the adjacent acupuncture point, but it was still appropriate for the volunteers to be free of needle sensation. To ensure the accuracy of the test, one acupuncturist was fixed to operate.
3. In Table 1, nine volunteers participated in the test of ST 36 point, nine volunteers participated in the test of SP 6 point, nine volunteers participated in the test of GB 39 point, 11 volunteers participated in the test of GB 37 point, and 10 volunteers participated in the test of KI 9 point. It is worth clarifying that when testing the above five acupuncture points, the same volunteer could participate in the testing of different acupuncture points and their nonacupuncture points. The power values for the nonacupuncture points were taken as the average of the four points tested. The same acupuncture point and its nonacupuncture points can vary greatly in the data tested by the instrument due to the different body sizes of the volunteers. At the same time, the data from the instrument also vary greatly from point to point and from nonpoint to nonpoint. For the sake of data authenticity, we present the complete data in the table. In Table 2, the ratio of the power values of the acupuncture points is measured by each volunteer to the power values of the nonacupuncture points. A value greater than “0” indicates that the power value of the acupuncture point is higher than the power value of the nonacupuncture point; a value less than “0” indicates that the power value of the acupuncture point is lower than the power value of the nonacupuncture point. The average power value in the last row is the percentage of all power values measured at the point compared to all power values at the nonacupuncture points.

Our current experiment with five acupuncture points showed that the power of acupuncture points differs from nonacupuncture points, and in the next experiment, we are planning to increase the number of acupuncture points to 10 to continue to take evidence and demonstrate the difference between acupoints and nonacupoint. It is a strong proof of the theory of Chinese medicine and one of the foundations for further research.

## 5. Conclusions

There are two main findings in the article. First, it was shown that bioelectricity signals of ST 36, SP 6, GB 37, GB 39, and KI 9 have high power values; second, we found the most significant differences in the frequency range of 0–2 Hz. Also, we could evaluate the power spectrum and power of acupuncture points and how they differ from nonacupuncture points. In addition, we can explore the specificity of the bioelectricity signals of acupuncture points in the future.

## Figures and Tables

**Figure 5:**
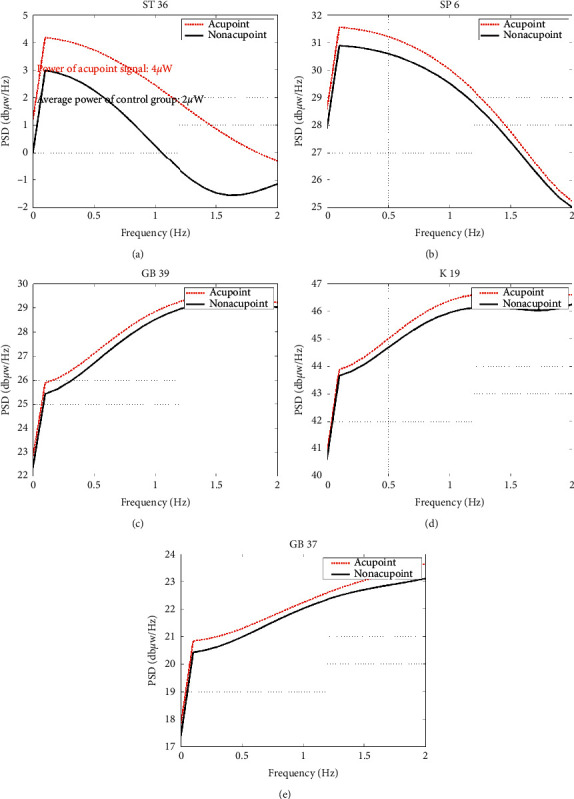
Average power spectrum of acupoints and control points.

**Table 1:** Specific measurement values.

Name	ST 36		SP 6			
Num	Acupoint power value	Nonacupoint power value	Acupoint power value		Nonacupoint power value	
1	1135.70	1106.30	3403.90		3265.60	
2	882.03	848.60	378.62		344.47	
3	1925.30	1853.80	893.47		849.89	
4	1474.80	1422.70	1892.60		1633.20	
5	1194.00	1127.50	125910.00		121480.00	
6	1949.50	1850.30	43357.00		39865.00	
7	2195.40	2103.70	372670.00		348470.00	
8	685.83	663.23	153120.00		155470.00	
9	2745.40	2588.20	88900.00		85157.00	

Name	GB 39		GB 37		KI 9	
Num	Acupoint power value	Nonacupoint power value	Acupoint power value	Nonacupoint power value	Acupoint power value	Nonacupoint power value

1	1468.40	1397.90	327.34	304.29	105550.00	97348.00
2	2212.90	2126.00	923.11	921.77	137760.00	93227.00
3	1353.40	1269.20	489.34	480.80	276340.00	271600.00
4	3030.80	2960.90	474.53	478.75	124010.00	118280.00
5	187040.00	177190.00	125.24	118.73	139560.00	134240.00
6	18194.00	17260.00	202.84	205.50	11833.00	11103.00
7	96560.00	91971.00	193870.00	188840.00	75489.00	69825.00
8	638580.00	612650.00	50471.00	48380.00	85692.00	82349.00
9	417290.00	407530.00	84470.00	83119.00	692280.00	662160.00
10			577420.00	556300.00	313710.00	271070.00
11			438300.00	415470.00		

**Table 2:** Percentage of difference between acupuncture and nonacupuncture points.

Name	Num	ST 36 (%)	SP 6 (%)	GB 39 (%)	GB 37 (%)	KI 9 (%)
Each volunteer point power value percentage higher than the nonacupoints	1	2.66	4.24	5.04	7.58	7.77
	2	3.94	9.91	4.09	0.15	32.33
	3	3.86	5.13	6.63	1.78	1.72
	4	3.66	15.88	2.36	−0.88	4.62
	5	5.90	3.65	5.56	5.48	3.81
	6	5.36	8.76	5.41	−1.29	6.17
	7	4.36	6.94	4.99	2.66	7.50
	8	3.41	−1.51	4.23	4.32	3.90
	9	6.07	4.40	2.39	1.63	4.35
	10				3.80	13.59
	11				5.49	
					
Average		4.36	6.38	4.52	2.79	8.58

## Data Availability

The data of the figures used to support the findings of this study are included in the article. The data of the tables used to support the findings of this study are available from the corresponding author upon request.
